# Effects of adenosine triphosphate and reduced glutathione on biochemical, histopathological and immunofluorescence alterations associated with atezolizumab-induced cardiac tissue injury

**DOI:** 10.3389/fphar.2026.1914819

**Published:** 2026-09-04

**Authors:** Yesim Kaya Yasar, Esra Tuba Sezgin, Bahadir Suleyman, Renad Mammadov, Mustafa Ozkaraca, Ali Gungor, Taha Abdulkadir Coban, Halis Suleyman

**Affiliations:** 1 Department of Pharmacology, Faculty of Pharmacy, Karadeniz Technical University, Trabzon, Türkiye; 2 Anesthesia Program, Vocational School of Health Services, Erzincan Binali Yıldırım University, Erzincan, Türkiye; 3 Department of Pharmacology, Faculty of Medicine, Erzincan Binali Yıldırım University, Erzincan, Türkiye; 4 Department of Pathology, Faculty of Veterinary Medicine, Cumhuriyet University, Sivas, Türkiye; 5 Laboratory and Veterinary Health Program, Vocational School of Health Services, Osmaniye Korkut Ata University, Osmaniye, Türkiye; 6 Department of Biochemistry, Faculty of Medicine, Erzincan Binali Yıldırım University, Erzincan, Türkiye

**Keywords:** adenosine triphosphate, atezolizumab, cardiac injury, glutathione, inflammation, myocarditis, oxidative stress, rat

## Abstract

**Background:**

In this study, the potential protective effects of adenosine triphosphate (ATP) and glutathione (GSH) against atezolizumab-induced cardiac injury in rat heart tissue were investigated.

**Methods:**

A total of 24 male albino Wistar rats were used in the experiment. The animals were divided into four groups: healthy group adenosine triphosphate + atezolizumab (ATAZ), GSH + atezolizumab (GHAZ), and atezolizumab alone (ATZ). ATP was administered intraperitoneally at a dose of 4 mg/kg, while GSH was administered orally at a dose of 200 mg/kg for 7 days. Atezolizumab was administered intraperitoneally to the experimental groups at a dose of 10 mg/kg twice weekly. At the end of the experiment, malondialdehyde total glutathione (tGSH), superoxide dismutase catalase, interleukin-1β (IL-1β), and interleukin-6 (IL-6) levels in heart tissues were measured using biochemical methods. In addition, histopathological and immunofluorescence examinations were performed.

**Results:**

The results showed that oxidative stress and inflammation markers increased, whereas antioxidant parameters decreased in the atezolizumab-treated group. ATP and GSH administration significantly attenuated these changes and exerted protective effects on cardiac tissue.

**Conclusion:**

Overall, ATP and GSH ameliorated the biochemical, histopathological, and immunofluorescence changes associated with atezolizumab-induced cardiac injury in this experimental model.

## 1 Introduction

Atezolizumab is a monoclonal antibody that targets programmed cell death ligand 1 (PD-L1) and inhibits its interaction with programmed cell death protein 1 (PD-1) (Ameri et al., 2022). It has been approved by the U.S. Food and Drug Administration (FDA) for use as monotherapy or in combination with other chemotherapeutic agents in the treatment of various neoplastic diseases, including locally advanced or metastatic urothelial carcinoma, metastatic non-small cell lung cancer, extensive-stage small cell lung cancer, metastatic triple-negative breast cancer, and unresectable or metastatic hepatocellular carcinoma (Aleem and Shah, 2026). As with other PD-1/PD-L1 inhibitors, atezolizumab therapy is associated with immune-related adverse events that can affect virtually any organ system and may be severe or even fatal. In particular, cardiovascular toxicities such as myocarditis, pericarditis, arrhythmias, thrombosis, and vasculitis have been reported among the potentially life-threatening complications (Wang et al., 2018). Currently, no established cytoprotective agent is routinely used prophylactically to prevent atezolizumab-associated cardiac toxicity. In cases of ICI-associated myocarditis, management generally involves discontinuation of ICI therapy and early initiation of immunosuppressive treatment (Wu et al., 2023). Although immune checkpoint inhibitor (ICI)-associated cardiac injury in humans is generally considered to result from PD-1/PD-L1 blockade, it remains unclear to what extent this mechanism is reproduced in experimental rat models. Nevertheless, experimental administration of atezolizumab has been reported to increase oxidative stress and induce inflammatory tissue injury (Wu et al., 2023). Furthermore, atezolizumab monotherapy has been reported to increase intracellular Ca^2+^ and reactive oxygen species (ROS) levels in myocytes, while reducing ATP levels (Quagliariello et al., 2024). In addition, high-grade hypercalcemia has been reported in a subset of cancer patients treated with atezolizumab, although the underlying mechanism remains to be elucidated (Rafae et al., 2022; Izzedine et al., 2022). It is well established that a decrease in ATP levels leads to an increase in intracellular Ca^2+^ levels, induction of ROS production, and apoptosis, whereas a further decline in ATP levels results in a shift toward necrosis, an uncontrolled form of cell death (Bulut and Süleyman, 2024). ROS, as oxidants, play a critical role in the development of various types of tissue damage, whereas antioxidants are essential in their mitigation (Kisaoglu et al., 2013). Endogenous reduced GSH is one of the most important antioxidants (Forman et al., 2009). ATP is required for the enzymatic synthesis of endogenous GSH; therefore, a decrease in ATP levels directly leads to reduced GSH synthesis (Lu, 2009). GSH is a non-protein thiol molecule involved in cellular defense against oxidative stress and is widely distributed in all mammalian tissues (Lu, 2013). In addition, ATP has been reported to exert anti-inflammatory effects and to inhibit the formation of free radicals (Swennen et al., 2006). Current evidence suggests that the cardiotoxic effects of atezolizumab may be associated with cellular ATP depletion, leading to decreased GSH levels, increased intracellular Ca^2+^, and enhanced oxidative stress.

To the best of our knowledge, there are no studies in the literature investigating the protective effects of ATP and GSH against atezolizumab-induced cardiac injury. Therefore, the aim of the present study was to investigate the protective effect of ATP against possible atezolizumab-induced cardiac injury in rats and to evaluate its effects in comparison with GSH.

## 2 Materials and methods

### 2.1 Animals

A total of 24 male albino Wistar rats, weighing between 280 and 290 g, were used in the experiment. The animals were obtained from the Experimental Animals Application and Research Center of Erzincan Binali Yıldırım University. Prior to the experiment, the animals were housed in groups (n = 6) under controlled conditions, including a temperature of 21–22 °C, relative humidity of 30%–70%, and a 12 h light/12 h dark cycle, with free access to water and standard pellet diet. All experimental procedures were approved by the Local Ethics Committee for Animal Experiments of Erzincan Binali Yıldırım University (meeting date: 30 April 2026; meeting number: 2026/04) and were conducted in accordance with the ARRIVE guidelines and the European Directive 2010/63/EU on the protection of animals used for scientific purposes (Percie du Sert et al., 2020). The sample size (n = 6 per group) was determined based on a previous experimental study using a similar rat model and biochemical outcome measures (Erhan et al., 2025) and in accordance with ethical principles aimed at reducing the number of animals used. However, a formal *a priori* power analysis was not performed prior to the study.

### 2.2 Chemicals

The commercially available form of atezolizumab, Tecentriq (F. Hoffmann-La Roche, Switzerland; 1,200 mg/20 mL), used in the study, was obtained from the Oncology Clinic of Erzincan Binali Yıldırım University Mengücek Gazi Training and Research Hospital. ATP was commercially obtained from Sigma-Aldrich (United States). Reduced GSH (capsules containing 250 mg reduced glutathione, Solgar, United States) and Thiopental sodium (İE Ulagay, Turkey) were obtained commercially.

### 2.3 Experimental animals

The animals were divided into four groups: healthy group (HG), ATP + atezolizumab (ATAZ), GSH + atezolizumab (GHAZ), and atezolizumab alone (ATZ).

### 2.4 Experimental procedure

ATP (4 mg/kg) was administered intraperitoneally (i.p.) once daily for 7 days to the ATAZ group (n = 6), based on a previous experimental study demonstrating the protective effects of ATP against drug-induced tissue injury (Erhan et al., 2025), while GSH (200 mg/kg) was administered orally once daily for 7 days to the GHAZ group (n = 6), based on a previous experimental study demonstrating the protective effects of GSH against drug-induced tissue injury (Yurttancikmaz et al., 2024). The HG (n = 6) and ATZ (n = 6) groups received distilled water (1 mL/kg, i.p.) as the vehicle. One hour after the administration of ATP, GSH, or vehicle, atezolizumab was administered i.p. at a dose of 10 mg/kg to the ATAZ, GHAZ, and ATZ groups twice weekly (on days 1 and 4), based on a previously established *in vivo* experimental administration protocol (Rattanapisit et al., 2023). On day 7, the rats were sacrificed under high-dose anesthesia (50 mg/kg thiopental sodium), and heart tissues were collected. In the extracted tissues, levels of MDA, tGSH, SOD, catalase, IL-1β, and IL-6 were measured. The tissues were also examined using histopathological and double immunofluorescence methods. Biochemical, histopathological, and immunofluorescence findings obtained from the experiment were compared and evaluated among the groups.

### 2.5 Biochemical analyses

#### 2.5.1 Sample preparation

Tissue samples were weighed, cut into small pieces, rapidly frozen in liquid nitrogen, and homogenized using a mortar and pestle. The homogenates were prepared in phosphate-buffered saline (PBS; pH 7.4) at a ratio of 1:10 (w/v) and vortexed for 10 s. Subsequently, the samples were centrifuged at 10,000 rpm for 20 min at 4 °C, and the supernatants were collected. All samples were stored at −80 °C until further analysis.

#### 2.5.2 Tissue MDA, tGSH, SOD, and catalase analyses

The MDA, tGSH and SOD analysis in tissues were performed with rat Enzyme-Linked Immunosorbent Assay (ELISA) kits following the kit instructions (product numbers 10009055, 703002 and 706002, Cayman Chemical Company, respectively). The catalase was determined according to the method recommended by Goth (Goth, 1991). Determination of tissue protein levels was measured as described by Bradford (Bradford, 1976).

#### 2.5.3 Tissue IL-1β and IL-6 analyses

The concentrations of IL-1β (pg/mg protein) and IL-6 (pg/mg protein) were determined using commercial ELISA kits (YL Biont, Shanghai YL Biotech Co., Ltd., China; Rat IL1B ELISA Kit Cat no. YLA0030Ra, Rat IL6 ELISA Kit Cat no. YLA0031Ra) in accordance with the manufacturer’s instructions. Protein concentrations used for the normalization of IL-1β and IL-6 levels were determined using the Bradford method (Bradford, 1976), and cytokine concentrations were expressed as pg/mg protein. IL-1β and IL-6 are key pro-inflammatory cytokines involved in immune checkpoint inhibitor-associated cardiac inflammation and were selected because they are widely used markers of immune-mediated cardiac injury.

### 2.6 Histopathological examination

Heart tissue samples were fixed in 10% neutral buffered formalin and embedded in paraffin blocks. Sections of 5 μm thickness were obtained from the paraffin blocks, deparaffinized, and passed through a graded alcohol series (100%, 90%, and 70%), followed by staining with hematoxylin and eosin (H&E). The sections were evaluated microscopically in terms of histopathological findings. The observed microscopic changes were assessed semi-quantitatively as none (0), mild (1), moderate (2), and severe (3) (Gibson-Corley et al., 2013). For each animal, six randomly selected non-overlapping microscopic fields were evaluated at ×400 magnification by two investigators who were blinded to the treatment groups.

### 2.7 Double immunofluorescence staining

Sections of 5 μm thickness mounted on poly-L-lysine-coated slides were deparaffinized in xylene and rehydrated through graded alcohol series, followed by washing in PBS. Antigen retrieval was performed using antigen retrieval solution (citrate buffer, pH 7.4) at 800 W for 2 × 5 min. The sections were then washed twice in PBS for 10 min each and incubated in PBS/gelatin/Triton X-100 (0.25%) solution for 10 min. Subsequently, sections were blocked with 5% BSA for 1 h. After blocking, the sections were incubated overnight at +4 °C with primary antibody pairs consisting of monoclonal 3,3-dityrosine (3,3-DTY) (Jaica, Cat. no. MDT-020P) and polyclonal 4-hydroxynonenal (4-HNE) (Genetex, Cat. no. GTX01087), as well as monoclonal IL-1β (Santa Cruz, Cat. no. sc-52012) and polyclonal IL-6 (Affbiotech, Cat. no. DF6087), at a dilution of 1:200. Following PBS washes, the sections were incubated for 45 min with a secondary antibody cocktail prepared in 1% BSA, containing equal amounts of Goat Anti-Mouse FITC (ImmunoResearch, Cat. no. 115-095-003) and Anti-Rabbit Alexa Fluor 595 (Cell Signaling, Cat. no. 8889S) at a dilution of 1:100. After incubation, the sections were washed in PBS and then treated with 10 mM CuSO_4_/50 mM NH_4_Cl solution for 10 min. Following rinsing with distilled water, DAPI (4′,6-diamidino-2-phenylindole) was applied. The sections were examined using a fluorescence microscope system (Zeiss Axiolab 5, Axiocam 305 color camera, Colibri 3). During evaluation, immunopositivity of IL-6 and 4-HNE was visualized in red, whereas 3,3-DTY and IL-1β immunopositivity were visualized in green. The staining intensity was assessed semi-quantitatively using Zen Blue 3.1 software as none (0), mild (1), moderate (2), severe (3), and very severe (4). For each animal, six randomly selected, non-overlapping microscopic fields were evaluated at ×400 magnification by two investigators who were blinded to the treatment groups.

### 2.8 Statistical analysis

Biochemical data were expressed as mean ± standard deviation (SD). The normality of biochemical data was assessed using the Shapiro–Wilk test prior to statistical analysis. Differences among groups were analyzed using one-way analysis of variance (ANOVA), followed by Tukey’s *post hoc* test when a significant difference was detected. All statistical analyses were performed using SPSS for Windows version 18.0 (SPSS Inc., Chicago, IL, United States). A value of p < 0.05 was considered statistically significant. Histopathological and double immunofluorescence data were analyzed using SPSS version 20.0. Group comparisons were performed using the non-parametric Kruskal–Wallis test, and the Mann–Whitney U test was used to determine differences between individual groups. A value of p < 0.05 was accepted as statistically significant.

## 3 Results

### 3.1 Biochemical findings

#### 3.1.1 Tissue MDA and tGSH levels

**FIGURE 1:**
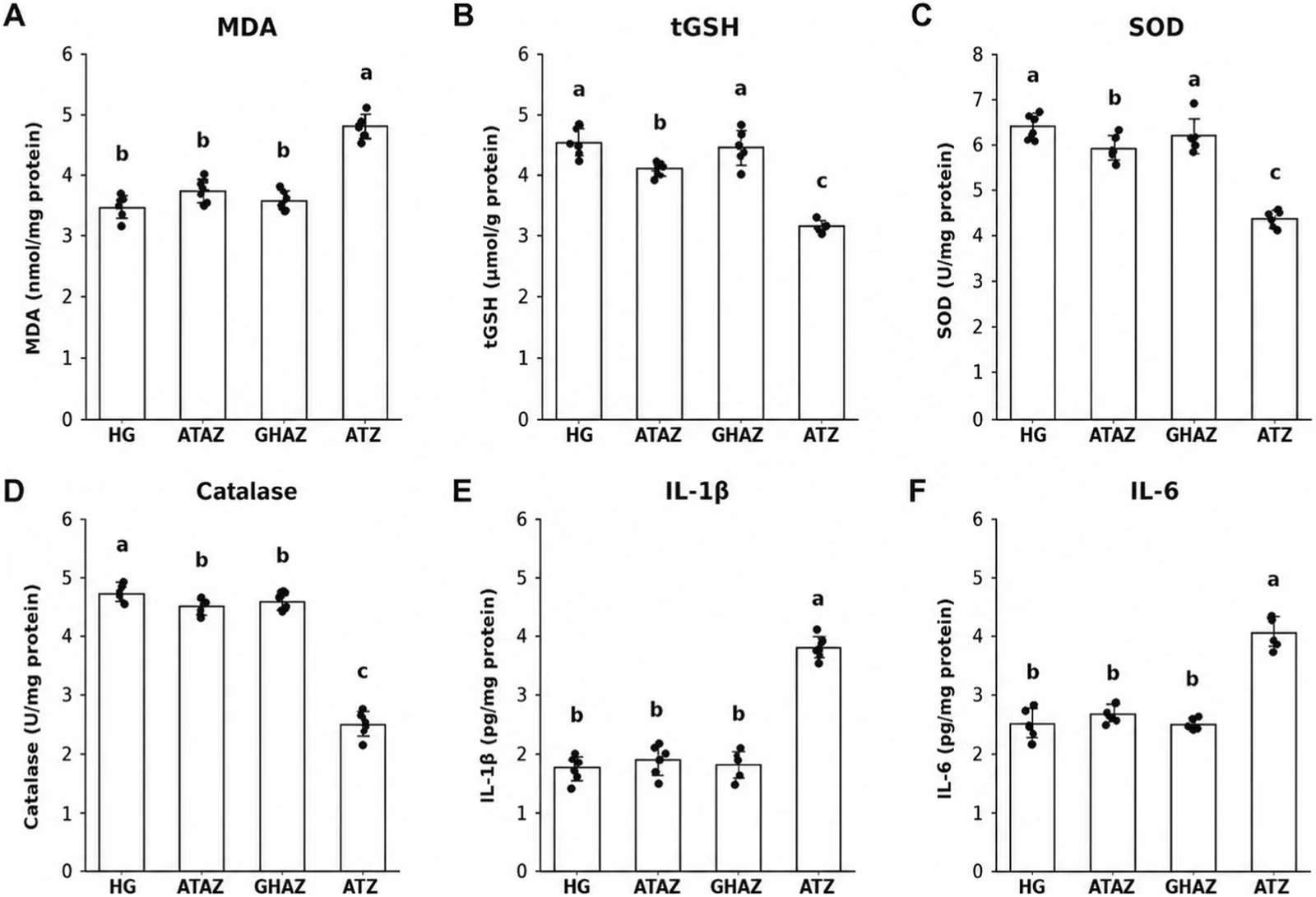
Oxidative stress, antioxidant enzyme activities, and inflammatory markers in tissue samples. **(A)** MDA, **(B)** tGSH, **(C)** SOD, **(D)** Catalase, **(E)** IL-1β, and **(F)** IL-6 levels in HG, ATAZ, GHAZ, and ATZ groups. Data are presented as mean ± SD (n = 6 per group). Different lowercase letters indicate statistically significant differences between groups (p < 0.05, one-way ANOVA followed by Tukey’s *post hoc* test). Abbreviations: HG, healthy group; ATAZ, ATP + atezolizumab group; GHAZ, GSH + atezolizumab group; ATZ, atezolizumab group.

As shown in Figure 1A; MDA levels differed significantly among the groups (p = 1.34 × 10^−10^), with the ATZ group showing higher levels than all other groups. No significant differences were observed among the remaining groups.

Similarly, tGSH levels showed a significant difference among the groups (p = 1.71 × 10^−10^). The ATZ group had lower tGSH levels compared to all other groups. Additionally, tGSH levels were higher in the GHAZ group than in the ATAZ group, while no difference was observed between the GHAZ and HG groups. The ATAZ group showed lower levels compared to the HG group (Figure 1B).

#### 3.1.2 Tissue SOD and catalase activities

SOD activity differed significantly among the groups (p = 4.67 × 10^−10^). The ATZ group exhibited significantly lower SOD activity compared to all other groups. No significant differences were observed among the remaining groups, except for a borderline difference between the ATAZ and HG groups (Figure 1C).

Catalase activity differed significantly among the groups (p = 1.37 × 10^−16^). The ATZ group showed significantly lower Catalase activity compared to all other groups. No significant differences were observed among the remaining groups, except for a difference between the ATAZ and HG groups (Figure 1D).

#### 3.1.3 Tissue IL-1β and IL-6 levels

IL-1β and IL-6 levels differed significantly among the groups (p < 0.001 for both). Both cytokines were significantly elevated in the ATZ group compared to all other groups, while no significant differences were observed among the remaining groups (Figures 1E,F).

### 3.2 Histopathological findings

**FIGURE 2:**
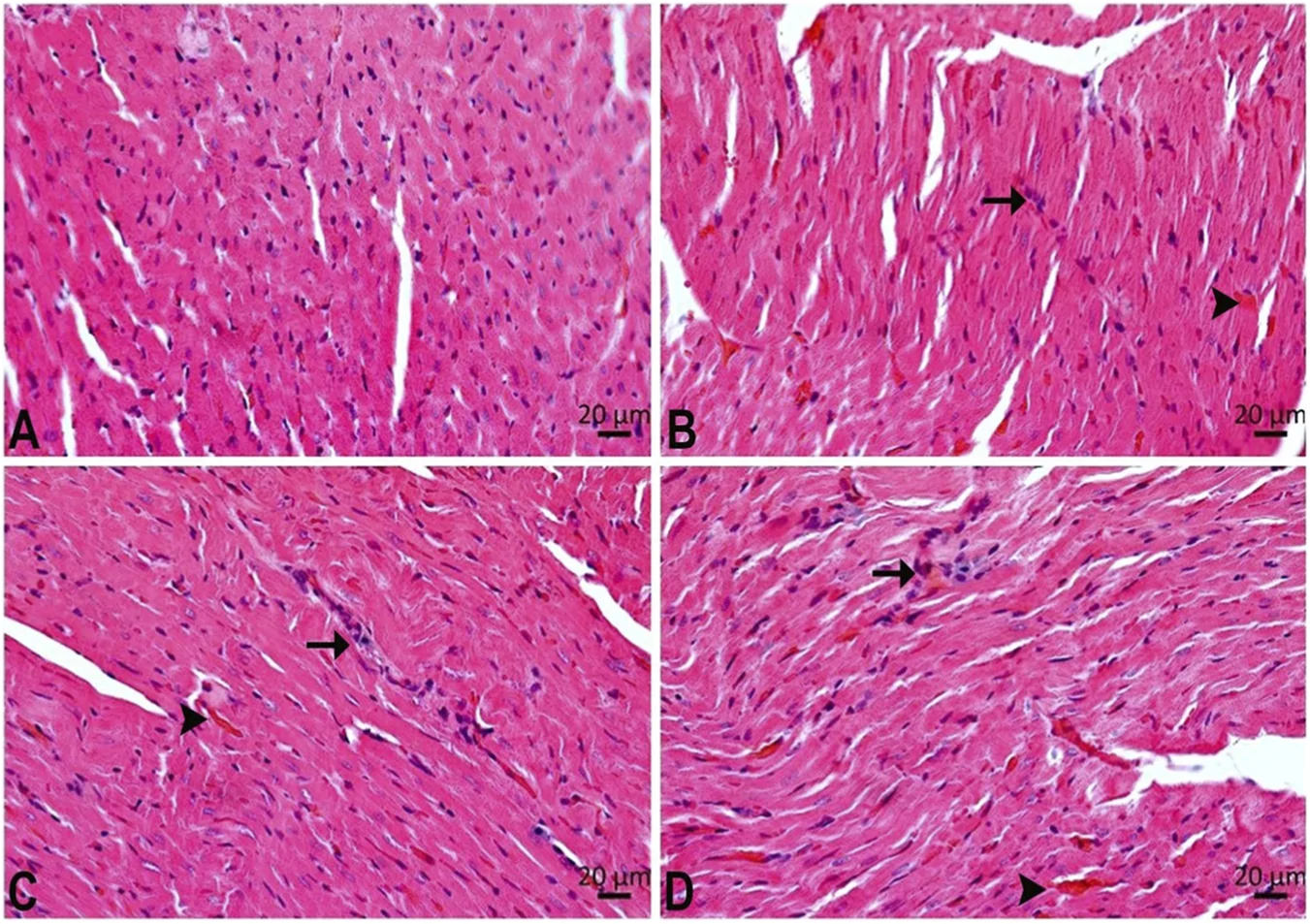
Histopathological findings in experimental groups. Representative images from each group are shown. **(A)** HG group showing normal histological architecture. **(B)** ATAZ group showing mild mononuclear cell infiltration (→) and hemorrhage (►). **(C)** GHAZ group showing moderate mononuclear cell infiltration (→) and hemorrhage (►). **(D)** ATZ group showing severe mononuclear cell infiltration (→) and hemorrhage (►). Sections were stained with H&E. Abbreviations: HG, healthy group; ATAZ, ATP + atezolizumab group; GHAZ, GSH + atezolizumab group; ATZ, atezolizumab group.

Histopathological examination revealed statistically significant differences among the experimental groups (Table 1). The HG group exhibited normal histological architecture. In the treatment groups, microscopic findings such as mononuclear cell infiltration in the interstitial areas and hemorrhage were observed. These findings were severe in the ATZ group, moderate in the GHAZ group, and mild in the ATAZ group (Figure 2).

**TABLE 1:** Histopathological scoring of mononuclear cell infiltration and hemorrhage in the experimental groups.

Groups	Mononuclear cell infiltration (score, median [min–max])	Hemorrhage (score, median [min–max])
HG	0 (0–0)^a^	0 (0–0)^a^
ATAZ	1 (0–1)^b^	1 (0–1)^b^
GHAZ	2 (2–3)^c^	2 (1–2)^c^
ATZ	3 (2–3)^d^	2 (2–3)^d^

Data are expressed as median (min–max). Statistical analysis was performed using the Kruskal–Wallis test followed by the Mann–Whitney U test for pairwise comparisons. Different superscript letters indicate statistically significant differences between groups (p < 0.05). Abbreviations: HG, healthy group; ATAZ, ATP + atezolizumab group; GHAZ, GSH + atezolizumab group; ATZ, atezolizumab group.

### 3.3 Double immunofluorescence findings

**FIGURE 3:**
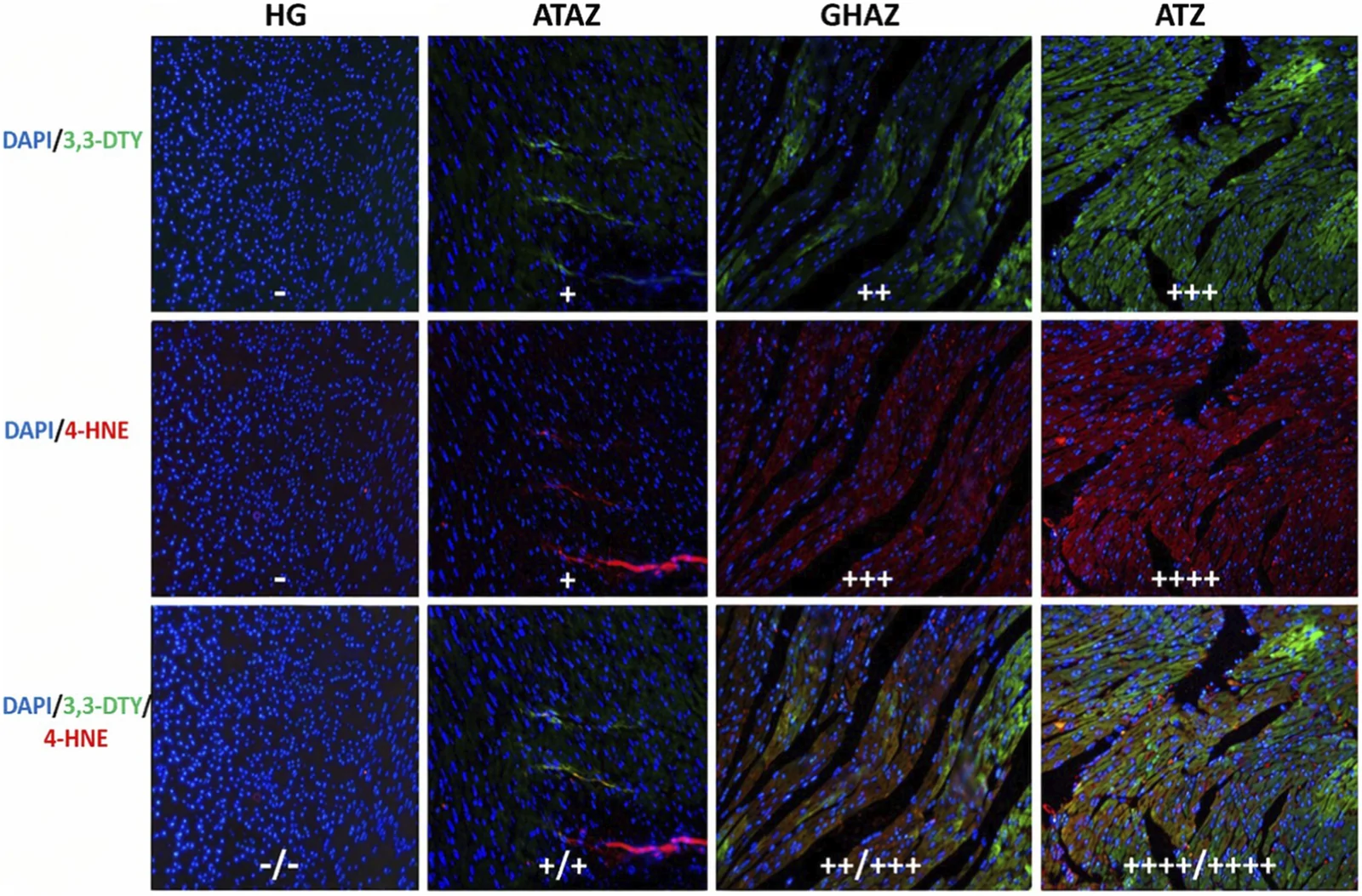
Immunofluorescence analysis of 3,3-DTY and 4-HNE expression in heart tissues of experimental groups: healthy (HG), mild (ATAZ), moderate (GHAZ), and severe (ATZ) (n = 6 per group). Fluorescence positivity was semi-quantitatively scored as absent (−), mild (+), moderate (++), severe (+++), and very severe (++++). 3,3-DTY was labeled with FITC, 4-HNE with Alexa Fluor 595, and nuclei were counterstained with DAPI. Statistical analysis was performed using the Kruskal–Wallis test followed by the Mann–Whitney U test for pairwise comparisons. Differences were considered statistically significant at p < 0.05. Abbreviations: HG, healthy group; ATAZ, ATP + atezolizumab group; GHAZ, GSH + atezolizumab group; ATZ, atezolizumab group.

**FIGURE 4:**
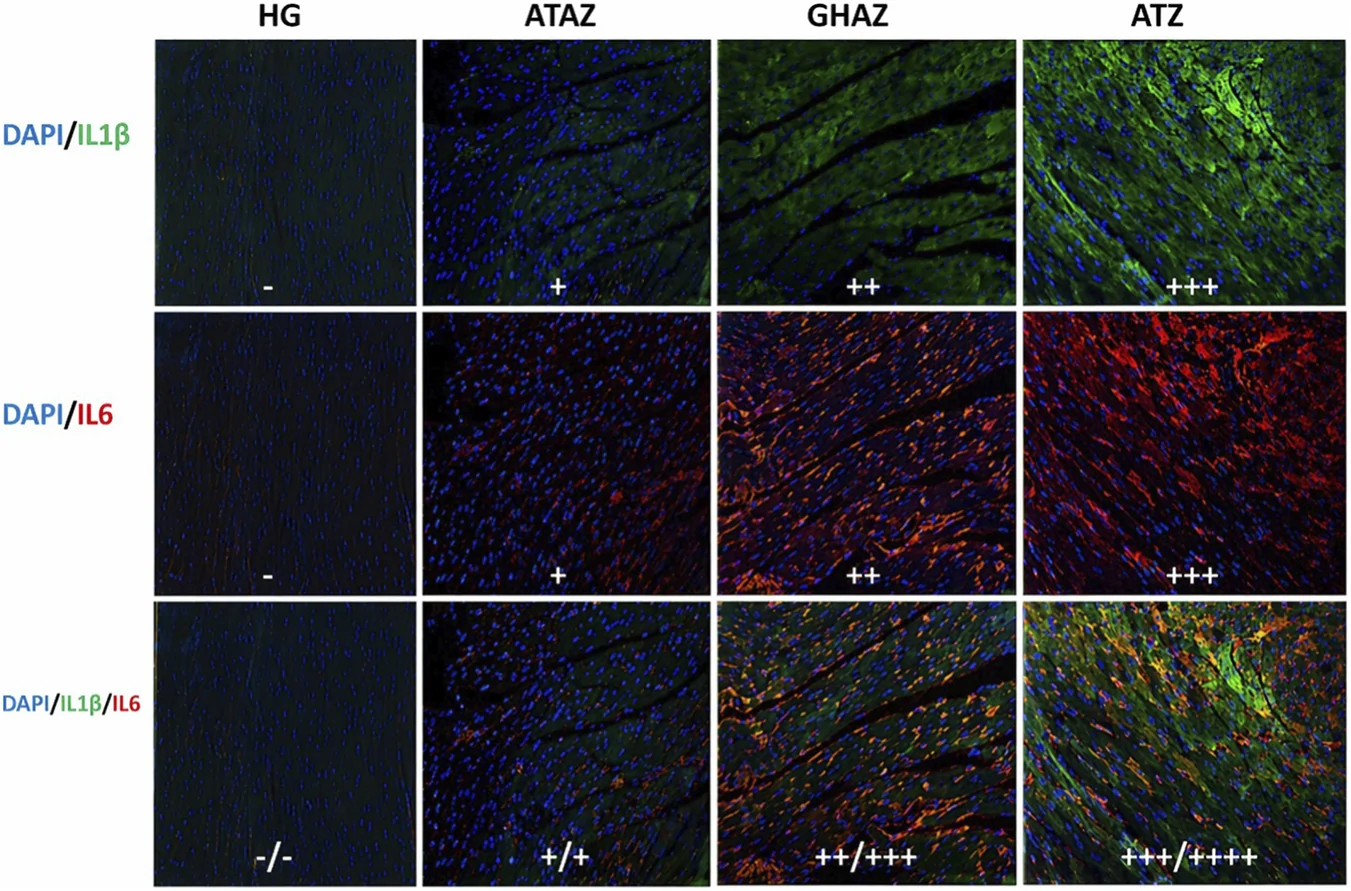
Immunofluorescence analysis of IL-1β and IL-6 expression in heart tissues of experimental groups: healthy (HG), mild (ATAZ), moderate (GHAZ), and severe (ATZ) (n = 6 per group). Fluorescence positivity was semi-quantitatively scored as mild (+), moderate (++), and severe (+++). IL-1β was labeled with FITC, IL-6 with Alexa Fluor 595, and nuclei were counterstained with DAPI. Statistical analysis was performed using the Kruskal–Wallis test followed by the Mann–Whitney U test for pairwise comparisons. Differences were considered statistically significant at p < 0.05. Abbreviations: HG, healthy group; ATAZ, ATP + atezolizumab group; GHAZ, GSH + atezolizumab group; ATZ, atezolizumab group.

In cardiac tissues, staining for 3,3-DTY, 4-HNE, IL-1β, and IL-6 revealed no sig-nificant immunoreactivity in the HG group, whereas varying degrees of positivity were observed in the treatment groups. 3,3-DTY immunoreactivity was mild in the ATAZ group, moderate in the GHAZ group, and very severe in the ATZ group. 4-HNE immunoreactivity was mild in the ATAZ group, severe in the GHAZ group, and very severe in the ATZ group. Immunoreactivity for IL-1β and IL-6 was mild in the ATAZ group, moderate in the GHAZ group, and severe in the ATZ group (Table 2; Figures 3, 4).

**TABLE 2:** Comparison of oxidative stress and inflammatory markers among experimental groups.

Groups	3,3 dityrosine (score, median [min–max])	4-HNE (score, median [min–max])	IL-1β (score, median [min–max])	IL-6 (score, median [min–max])
HG	0 (0–0)^a^	0 (0–0)^a^	0 (0–0)^a^	0 (0–0)^a^
ATAZ	1 (0–1)^b^	1 (0–1)^b^	1 (0–1)^b^	1 (0–1)^b^
GHAZ	2 (1–2)^c^	3 (2–3)^c^	2 (1–2)^c^	2 (1–2)^c^
ATZ	3 (2–3)^d^	4 (3–4)^d^	3 (2–3)^d^	3 (2–3)^d^

Data are expressed as median (min–max). Statistical analysis was performed using the Kruskal–Wallis test followed by the Mann–Whitney U test for pairwise comparisons. Different superscript letters (a–d) indicate statistically significant differences between groups (p < 0.05). Abbreviations: HG, healthy group; ATAZ, ATP + atezolizumab group; GHAZ, GSH + atezolizumab group; ATZ, atezolizumab group.

## 4 Discussion

In this study, the effects of ATP and GSH on biochemical, histopathological, and immunofluorescence indicators of atezolizumab-induced cardiac tissue injury were investigated. The obtained data showed that atezolizumab increased levels of oxidative stress markers, particularly MDA, while significantly decreasing antioxidant parameters such as tGSH, SOD, and catalase. Atezolizumab was also found to elevate proinflammatory cytokines, including IL-1β and IL-6. These biochemical alterations were accompanied by marked histopathological damage and increased immunofluorescence expression of oxidative stress and inflammatory markers.

Myocarditis is one of the immune-related adverse effects of atezolizumab; although rare, this toxicity can progress rapidly (Tokunaga et al., 2024). Although the mechanism of atezolizumab-induced cardiac injury has not yet been fully elucidated, it is thought to arise as a result of autoimmune mechanisms and the immune system response (Wu et al., 2023; Heymans et al., 2024). In addition, oxidative stress is considered one of the key mechanisms involved in the pathogenesis of cardiac injury (Xu et al., 2025). As is well known, oxidative damage occurs due to an imbalance between the production of ROS and their neutralization by the antioxidant defense system (Aranda-Rivera et al., 2022). One of the important markers of oxidative stress is MDA (Cordiano et al., 2023). In our study, the high MDA levels observed in the group treated with atezolizumab alone are consistent with previous studies reporting that atezolizumab causes oxidative damage (Liu et al., 2021). This finding suggests that atezolizumab may induce oxidative stress through lipid peroxidation (LPO). Atezolizumab also significantly decreased tGSH levels in cardiac tissue. GSH is a component of the non-enzymatic antioxidant system and plays a role in detoxification and the regulation of immune function (Pérez et al., 2020). In our study, the decrease in tGSH levels observed in the atezolizumab-treated group is consistent with reports indicating that it is associated with oxidative stress–mediated mechanisms (Mytilineou et al., 2002). In addition, some of the key enzymatic parameters of the antioxidant system are SOD and catalase. The decrease observed in SOD and catalase activities indicates a weakening of the antioxidant defense system and, according to the literature, suggests the presence of oxidative stress (Jomova et al., 2024). IL-1β and IL-6, which were found to be elevated in the cardiac tissues of atezolizumab-treated animals, are proin-flammatory cytokines involved in immune activation and tissue damage processes (Abbas et al., 2021). In humans, ICI-associated myocarditis is generally attributed to PD-1/PD-L1 blockade and subsequent T-cell activation (Wu et al., 2023). However, direct evidence demonstrating functional binding of atezolizumab to rat PD-L1 remains limited. Previous studies have demonstrated that atezolizumab is cross-reactive with murine PD-L1 (Magiera-Mularz et al., 2021), whereas its interaction with rat PD-L1 has not been fully characterized. Therefore, the present findings should be interpreted as evidence of atezolizumab-associated experimental cardiac injury rather than definitive proof of PD-1/PD-L1-mediated immune checkpoint blockade. Nevertheless, the observed increases in oxidative stress markers and pro-inflammatory cytokines indicate that atezolizumab induced a marked inflammatory response in this experimental model.

ATP, whose protective effect against atezolizumab-induced cardiac injury was investigated, is the primary energy source for all cellular functions (Huang et al., 2022). There is a bidirectional relationship between oxidative stress and ATP depletion; while oxidative stress reduces ATP production, energy deficiency in turn triggers an increase in ROS, thereby exacerbating cellular damage (Wu et al., 2022). In our study, ATP significantly prevented the atezolizumab-induced increase in MDA levels and decreases in tGSH levels, SOD activity, and catalase activity. This finding is consistent with the literature indicating that ATP can prevent oxidative stress and exert protective effects against cardiac damage (Lian et al., 2016). Similarly, Ozgodek et al. (2026) demonstrated that the impaired redox balance in oxidative damage was restored with ATP administration (Ozgodek et al., 2026). In addition to oxidative stress, inflammation also plays a critical role in the development of cardiovascular damage (Manful et al., 2025). In a study by Swennen et al., it was emphasized that ATP suppresses the inflam-matory response (Swennen et al., 2006). In our experimental results, the suppression of the atezolizumab-induced increase in IL-1β and IL-6 levels by ATP suggests that purinergic signaling may shift toward an adenosine-mediated anti-inflammatory direction (Antonioli et al., 2013). Furthermore, extracellular ATP mediates its effects primarily through purinergic receptors, which play a critical role in regulating oxidative stress responses and modulating inflammation (Di Virgilio et al., 2018). Similarly, Esenulku et al. (2025) reported that elevated inflammatory cytokine levels in drug-induced tissue damage were alleviated through ATP administration (Esenulku et al., 2025). In our study, the increase in oxidant markers and inflammatory cytokine levels, along with the decrease in antioxidant levels following atezolizumab administration, suggests possible drug-induced cardiac damage. Furthermore, these findings suggest that ATP attenuates biochemical and histopathological evidence of atezolizumab-induced cardiac tissue injury and may contribute to cardioprotection. Unlike our previous studies investigating the protective effects of ATP in other drug-induced toxicity models, the present study focuses on atezolizumab-associated cardiac injury. In this model, immune-mediated inflammatory mechanisms are believed to play an important role in addition to oxidative stress. Therefore, the potential cardioprotective effects of ATP may be related not only to the attenuation of oxidative stress but also to the modulation of inflammatory responses associated with immune checkpoint inhibitor therapy. Mitochondrial dysfunction resulting from impaired ATP production and increased ROS generation may also contribute to atezolizumab-induced cardiac injury. However, mitochondrial function was not evaluated in the present study; therefore, this possibility could not be directly demonstrated and warrants further investigation in future studies.

GSH, whose effect against atezolizumab-induced cardiac injury was investigated, is an important endogenous antioxidant (Forman et al., 2009). GSH plays a key role in providing cellular protection against oxidative stress (Lu, 2013). It is a major antioxidant that reduces oxidative stress by neutralizing ROS and suppresses the NF-κB-mediated inflammatory response. Due to these properties, increased GSH levels are considered to have a protective effect against cardiac damage (Tan et al., 2023). In our study, the shift of the oxidant–antioxidant balance toward antioxidants and the attenuation of increased inflammatory cytokine levels following GSH administration are consistent with the aforementioned literature. Previous studies have shown that GSH administration inhibits the increase in MDA and IL-6 levels, as well as the decrease in SOD and catalase activities; these findings demonstrate the antioxidant and anti-inflammatory effects of GSH (Somade et al., 2020; Supriyadi et al., 2023). When the GSH- and ATP-treated groups were compared, no statistically significant differences were observed in MDA, SOD, catalase, IL-1β, or IL-6 levels. However, because ATP and GSH were administered at different doses and via different routes, and pharmacokinetic equivalence was not evaluated, direct comparisons between the two agents should be interpreted with caution. The significantly higher tGSH level observed in the GSH-treated group compared with the ATP-treated group may be attributed to exogenous GSH administration. Because ATP-alone and GSH-alone groups were not included in the present study, it cannot be excluded that part of the observed biochemical improvements may be attributable not only to their protective effects against atezolizumab-induced injury but also to the intrinsic antioxidant and anti-inflammatory properties of ATP and GSH. Therefore, although our findings suggest that ATP and GSH attenuate atezolizumab-associated cardiac injury, these findings should be interpreted with the understanding that the independent effects of these agents in the absence of atezolizumab could not be evaluated.

Our histopathological findings were not entirely consistent with the biochemical data. Although no significant differences were observed between the ATP- and GSH-treated groups for most biochemical parameters, histopathological evaluation revealed less mononuclear cell infiltration and hemorrhage in the ATP-treated group. This discrepancy may be attributable to the different biological characteristics of the evaluated endpoints. Biochemical analyses performed on whole-tissue homogenates reflect the overall oxidative and inflammatory status of the tissue, whereas histopathological examination may reveal more localized structural alterations. Moreover, semi-quantitative histopathological scoring is inherently more variable than biochemical measurements. Therefore, the observed differences between the biochemical and histopathological findings should be interpreted with caution. Nevertheless, previous studies have shown that atezolizumab induces inflammatory tissue injury and histopathological alterations (Liu et al., 2023; Aschauer et al., 2022). ATP has been reported to preserve myocardial architecture in cardiac injury (Lian et al., 2016), whereas GSH has also been shown to attenuate oxidative stress, suppress inflammation, and preserve tissue integrity (Tan et al., 2023; Negm et al., 2025). Collectively, our findings suggest that both agents exert protective effects, although localized histopathological alterations appeared to be less pronounced in the ATP-treated group.

Although our biochemical findings did not fully align with our histopathological results, our immunofluorescence findings were consistent with the histopathological observations. 3,3-DTY is an indicator of protein oxidation (Davies, 2016), while 4-HNE is a marker of LPO (Ayala et al., 2014). The increase in both markers observed in the group treated with atezolizumab alone supports that it induces oxidative stress and cellular damage in the tissue (Liu et al., 2021). In terms of inflammatory components, the high levels of immunoposi-tivity for IL-1β and IL-6 observed in the atezolizumab-treated group indicate severe oxidative damage and inflammation (Magiera-Mularz et al., 2021). Low levels of positivity in the ATP group indicate limited damage, whereas moderate expression in the GSH group suggests a partial protective effect. The literature also indicates that both ATP, by maintaining cellular energy balance (Tan et al., 2023), and GSH, through its strong antioxidant properties, suppress inflammation and oxidative damage (Supriyadi et al., 2023).

This study has several limitations. First, the use of an experimental animal model restricts the direct translation of the findings to clinical settings. In addition, only male rats were included in the present study to minimize hormonal variability. Therefore, sex-related differences in atezolizumab-induced cardiac injury and in the potential cardioprotective effects of ATP and GSH could not be evaluated. The relatively short duration of the study limited the evaluation of the long-term cardiotoxic effects of atezolizumab, as well as the potential protective roles of ATP and GSH. The absence of ATP-alone and GSH-alone groups also prevented discrimination between the intrinsic biological effects of these agents and their specific protective effects against atezolizumab-induced injury. Therefore, baseline antioxidant and anti-inflammatory effects independent of atezolizumab cannot be excluded. In addition, the short-term intraperitoneal atezolizumab model used in this study may not fully reflect the clinical pattern of atezolizumab-induced cardiotoxicity. Therefore, the findings should be interpreted as outcomes of an acute/subacute experimental model of cardiac injury. In addition, molecular analyses regarding the mechanisms of action of ATP and GSH were not performed, and ATP levels in cardiac tissue were not measured. Furthermore, serum ATP and GSH levels, as well as cardiac tissue GSH levels, were not assessed; therefore, target engagement could not be directly confirmed. Future pharmacokinetic studies including direct measurements of serum and tissue ATP/GSH levels are warranted and would provide valuable insight into the bioavailability and efficacy of these agents. A formal *a priori* power analysis was not performed, and the relatively small sample size should be considered a limitation of the present study. The use of whole-heart sections and the consequent inability to evaluate potential regional differences in cardiac injury represent another limitation of the present study. In addition, no specific inhibitors were used during tissue sample preparation, which may have affected the stability of MDA and cytokines during sample processing and should be considered a methodological limitation of the study. Furthermore, the inflammatory infiltrate was evaluated morphologically but was not characterized using specific immune cell markers. Future studies employing immunohistochemical characterization of infiltrating immune cells will allow a clearer understanding of the mechanisms underlying the development of atezolizumab-induced cardiac injury. Also, the effects of endogenous ATP via purinergic signaling pathways were not evaluated, which limits a comprehensive understanding of the cardioprotective mechanisms of these agents. Additionally, future studies incorporating cardiac function assessments (such as Echocardiography parameters), serum troponin measurements, and other clinically relevant biomarkers are warranted to further clarify the potential cardioprotective effects of ATP and GSH against atezolizumab-induced cardiac injury. Moreover, the inherent instability of exogenous ATP and its rapid degradation by enzymes such as CD39 and CD73 may limit its bioavailability, which should be taken into consideration when interpreting the findings.

## 5 Conclusion

In conclusion, the findings of this experimental study suggest that atezolizumab administration is associated with increased oxidative stress, inflammation, and cardiac tissue injury in rats. ATP and GSH treatment attenuated biochemical, histopathological, and immunofluorescence evidence of cardiac tissue injury in this experimental rat model. Although both agents reduced tissue-level indicators of injury, ATP showed a greater degree of improvement in several evaluated parameters. These findings indicate that modulation of oxidative stress and inflammation may contribute to the observed protective effects; however, the underlying mechanisms remain to be clarified. Given the limitations of the present study, including the experimental model and the absence of mechanistic, pharmacokinetic, and dose–response evaluations, the results should be considered preliminary. The observed improvements were demonstrated at the tissue level. Future experimental and clinical studies including cardiac functional assessments, serum cardiac biomarkers, and mechanistic analyses are required to de-termine whether these tissue-level improvements translate into functional cardioprotection.

## Data Availability

The original contributions presented in the study are included in the article/Supplementary Material, further inquiries can be directed to the corresponding author.
