# Supplementary material for: Effects of adenosine triphosphate and reduced glutathione on biochemical, histopathological and immunofluorescence alterations associated with atezolizumab-induced cardiac tissue injury
Source: Front Pharmacol. 2026 Sep 4;17:1914819. doi: 10.3389/fphar.2026.1914819 (PMC13585631; doi:10.3389/fphar.2026.1914819)
Supplement: Supplementary file 1 [file Table1.docx]

**Supplementary Table S1**. One-way ANOVA results for biochemical parameters.

| **Parameter** | **F** | **df** | **p** |
| --- | --- | --- | --- |
| MDA (nmol/mg protein) | 66.814 | 3,20 | 1.34 × 10⁻¹⁰ |
| tGSH (µmol/g protein) | 65.046 | 3,20 | 1.71 × 10⁻¹⁰ |
| SOD (U/mg protein) | 58.154 | 3,20 | 4.67 × 10⁻¹⁰ |
| CAT (U/mg protein) | 286.208 | 3,20 | 1.37 × 10⁻¹⁶ |
| IL-1β (pg/mg protein) | 120.471 | 3,20 | 5.68 × 10⁻¹³ |
| IL-6 (pg/mg protein) | 90.210 | 3,20 | 8.53 × 10⁻¹² |
